# Habitat quality influences trade-offs in animal movement along the exploration–exploitation continuum

**DOI:** 10.1038/s41598-023-31457-3

**Published:** 2023-03-24

**Authors:** Joshua B. Smith, David A. Keiter, Steven J. Sweeney, Ryan S. Miller, Peter E. Schlichting, James C. Beasley

**Affiliations:** 1 Savannah River Ecology Laboratory, University of Georgia, P.O. Drawer E., Aiken, SC 29802 USA; 2 D.B. Warnell School of Forestry and Natural Resources, University of Georgia, 180 East Green St., Athens, GA 30602 USA; 3 United States Department of Agriculture, Animal and Plant Health Inspection Service, Veterinary Services, Center for Epidemiology and Animal Health, 2150 Centre Avenue, Fort Collins, CO 80526 USA; 4 Present Address: Oregon Department of Fish and Wildlife, 1401 Gekeler Ln, La Grande, OR 97850 USA

**Keywords:** Animal migration, Conservation biology, Invasive species

## Abstract

To successfully establish itself in a novel environment, an animal must make an inherent trade-off between knowledge accumulation and exploitation of knowledge gained (i.e., the exploration–exploitation dilemma). To evaluate how habitat quality affects the spatio-temporal scale of switching between exploration and exploitation during home range establishment, we conducted experimental trials comparing resource selection and space-use of translocated animals to those of reference individuals using reciprocal translocations between habitat types of differing quality. We selected wild pigs (*Sus scrofa*) as a model species to investigate hypotheses related to the movement behavior of translocated individuals because they are globally distributed large mammals that are often translocated within their introduced range to facilitate recreational hunting. Individuals translocated to higher quality habitat (i.e. higher proportions of bottomland hardwood habitats) exhibited smaller exploratory movements and began exploiting resources more quickly than those introduced to lower quality areas, although those in lower-quality areas demonstrated an increased rate of selection for preferred habitat as they gained knowledge of the landscape. Our data demonstrate that habitat quality mediates the spatial and temporal scale at which animals respond behaviorally to novel environments, and how these processes may determine the success of population establishment.

## Introduction

Animals are exposed to novel environments through natural and anthropogenic processes: natal dispersal, dispersal beyond their native range (e.g. Eurasian waterfowl in North America)^1^, passive transport by environmental conditions (e.g. terrestrial animals carried by ocean currents)^2^, accidental introduction (e.g., marine organisms transported in ship ballast)^3^ or deliberate translocation for conservation (e.g., reintroductions or population augmentation of endangered and game species)^4^ or recreational purposes (e.g., desired angling species)^5^. To successfully establish in a novel environment, appropriate resources and niche space must be available, and an animal must gain sufficient knowledge of the distribution and availability of these resources to allow its survival and reproductive success. However, there is an inherent and well-recognized trade-off between knowledge accumulation and exploitation of the knowledge gained, referred to as the exploration–exploitation dilemma^6,7^.

The cost of gaining knowledge through exploration can outweigh the benefit of exploiting short-term opportunities, or vice-versa, and thus optimal strategies should balance present and future benefits while accounting for resource heterogeneity in time or space^8^. It has been proposed the optimal trade-off between exploration and exploitation of habitats will vary depending upon the life-stage of an animal (e.g., it is sub-optimal to invest in gaining new information near the end of life); this pattern may also be evident in animals introduced to novel environments, as dispersal events essentially represent a reversion in knowledge levels^9^. Examination of fine-scale spatial–temporal patterns, such as speed and distance travelled, may allow scientists to identify phases of an animal’s establishment in a novel landscape to determine the period over which the animal is primarily exploring versus exploiting their environment^10^. Research suggests memory-based foraging is linked to the establishment and use of animal home ranges, which generally constitute a restricted portion of the total available landscape^11^. Although other factors can affect home range size (e.g., mating system, sex, type of forager)^12^, greater evaluation of spatial patterns observed as an animal establishes a home range in a novel environment is necessary to facilitate conservation and management programs.

Based on the exploration–exploitation dilemma, knowledge accumulation or exploration by an animal should be highest immediately following its introduction to a novel environment^9^. At this stage, the animal may live off energy reserves and explore the landscape to identify resources required for survival, and this exploration requires reduced exploitation of identified resources. Corroborating this hypothesis, multiple studies provide evidence of increased movements by animals immediately following translocation, e.g.,^13–16^. Once an animal attains familiarity with locations of sufficient resources in the novel environment, it is likely to begin exploiting these resources, and exhibiting reduced exploratory behavior. Eventually, movement patterns should resemble those of resident animals of the same species under similar conditions. In addition, research on translocations suggests habitat quality and competition may influence spatial patterns exhibited by animals in novel environments. For example, Frair et al*.*^17^ found site fidelity of translocated elk (*Cervus elaphus*) was related to patch forage quality surrounding the release site. Therefore, it might be expected that in areas where high quality habitat is present and readily accessible, animals will locate and map necessary resources in their novel environment more quickly and occupy a smaller home range due to the proximal availability of resources. Additionally, density of conspecifics or other species with overlapping niche space are likely to influence post-release behavior^18,19^. Nonetheless, as an animal accumulates spatial and temporal knowledge of the distribution of resources within the novel landscape following introduction, aspects of animal movement such as home range size, average daily distance moved, and resource selection should also change.

Previous studies have presented broad comparisons of home range size between translocated and resident animals^20–23^, but rarely are changes in home range size and movement patterns described at a fine temporal scale (e.g., daily or weekly movements post translocation), allowing elucidation of processes by which animals colonize new areas (but see^10,24^). Knowledge of fine-scale patterns in movement by animals in novel environments is necessary as a foundation for greater understanding of the mechanisms of species establishment^25^, and therefore has implications for improving existing exploration–exploitation theory and the conservation and management of wildlife populations.

In this study, we used a 7-day moving window to assess a suite of movement metrics (i.e., daily distance traveled, use area, and net squared displacement) to evaluate the processes by which animals establish a home range in novel areas to which they are translocated. We selected wild pigs (*Sus scrofa*) as a model species to investigate hypotheses related to the movement behavior of translocated individuals because they are a globally distributed large mammal that is considered an invasive species in many areas and expanding in range due to frequent translocations^26^. In regions where they are non-native, wild pigs are often translocated to facilitate recreational hunting opportunities, which is a primary mechanism contributing to the extensive range expansion of this species in their introduced range^27,28^. Previous research suggests pigs have well-developed spatial memories^29^, which likely facilitates their ability to successfully establish populations when introduced to a novel environment. Once introduced, wild pigs cause extensive ecological and economic impacts, and thus information is critically needed on post-release movements to facilitate management programs^30^. Although generalist foragers, wild pigs are heavily dependent on wetland and riparian habitats for foraging, resting, and thermoregulation^31–33^. Thus, riparian areas and bottomland hardwood swamps are considered high quality habitats for wild pigs, particularly relative to pine-dominated forests, and the distribution of these preferred habitats has a strong influence on wild pig movements^34^. Thus, the composition and configuration of bottomland hardwood forests, wetlands, and riparian areas within the landscape likely plays an important role in the movement behavior of translocated wild pigs or those dispersing into novel areas.

We hypothesized, based upon current exploration–exploitation theory, that translocated wild pigs would exhibit extensive exploratory movements (relative to typical movements for the species) following release into a novel environment (exploration phase). Subsequently, during the exploitation phase, we predicted these patterns would resemble those of established resident animals as evidenced by various attributes of movement such as home range size, daily movement distances, and resource selection. To test these predictions, we conducted experimental translocation trials complete with reverse translocations and control individuals, where we translocated female wild pigs from high quality habitat to low quality habitat and vice versa and compared movement patterns and resource selection of each experimental group to those of reference females to evaluate whether patterns of movement are mediated by habitat quality. We predicted translocated individuals would show reduced movements when introduced into higher quality habitat (bottomland hardwoods) compared to relatively low-quality habitat (upland pines), as exploitation of resources should require less travel and exploration in areas with abundant high quality habitat^35^. Similarly, we predicted animals translocated to higher quality habitat would take less time to exhibit movement patterns similar to resident animals than those translocated to lower quality habitat.

## Methods

### Data collection

We conducted this research on the Savannah River Site (SRS), a 780 km National Environmental Research Park on the border of South Carolina and Georgia, USA. Approximately 68% of habitat on the SRS consists of upland pine forest containing interspersed riparian areas, with another 22% comprised of bottomland hardwood forest, and the remaining areas consisting of open water, shrublands, industrial areas, and grasslands^36^. Although wild pigs occur throughout both upland pine and bottomland hardwood habitats on the SRS^37^, movements are often concentrated within bottomland hardwood and other riparian habitats^33,38^. Upland pine forests on the SRS are dominated by loblolly pine (*Pinus taeda*), long-leaf pine (*P. palustris*), and slash pine (*P. elliotii*) and are managed by the USDA Forest Service for timber and wildlife habitat^36^. Bottomland hardwood forests incorporate swamp and wetlands and are dominated by oaks (*Quercus *spp.), tulip-poplars (*Liriodendron tulipifera*), and other hardwood species^36^.

We trapped breeding-age female wild pigs in baited corral and box traps in upland pine and bottomland hardwood habitat from June 2014 to July 2016 (Table S1). We used a dart rifle (X-Caliber, Pneu-Dart Inc., Pennsylvania, USA) to anesthetize pigs using a combination of Telazol^®^ (4.4 mg/kg; MWI Veterinary Supply, Idaho, USA) and Xylazine (2.2 mg/kg; Wildlife Pharmaceuticals Inc., Colorado, USA). We recorded sex, assessed age through examination of tooth eruption, and collected morphological measurements from each animal. We collared pigs with a 3000S Global Positioning System (GPS) collar (Lotek Wireless Inc., Ontario, Canada) or a GPS Plus X Collar (Vectronic Aerospace GmbH, Berlin, Germany). Lotek 3000S GPS collars were programmed to take locations every 2 h, while GPS Plus X collars were programmed to collect locations at 15-min intervals for the first 6 weeks post capture, at which point we reprogrammed them to take locations at 1-h intervals. Anesthetized pigs were either reversed with Yohimbine (0.15 mg/kg) at their capture site and released to serve as reference animals or were transported while anesthetized to a novel release site to serve as translocated animals. South Carolina laws regarding the transportation of captured wild pigs limited translocations to within the boundary of the SRS property. Nonetheless, given the large area encompassed by the SRS we were able to translocate pigs at least 8 km from their point of capture, which is greater than the 95% credible interval for wild pig home range size in North America^39^ and greater than most reported dispersal events^40^. Additionally, although capture and translocation efforts were restricted to inside the SRS, wild pigs could move outside of the SRS boundaries. Among translocated animals, individuals captured in upland pine (low quality) habitat were translocated to bottomland hardwood (high quality) habitat and vice-versa. To assess overall habitat quality at each release site, we estimated the utilization distribution (UD) for each resident wild pig using kernel density estimation from the R package adehabitatHR^41^. The 95% isopleth of UD’s was used to define the home range size for each animal. We then buffered release sites by the average home range size (8.3 km^2^) and calculated percent upland pine and bottomland hardwood for each release habitat type. For wild pigs released in our classified bottomland hardwood habitat, 26% of the area contained bottomland hardwood while upland pine constituted 44%. Percentages for animals released into upland pine was 21% and 71% for bottomland hardwood and upland pine, respectively. Additional information on study species, study site, and data collection can be found in supplemental material. Additionally, to assess social integration of translocated wild pigs with local groups of wild pigs, in 2016 we placed baited white-flash trail cameras (Reconyx PC900 Hyperfire, Reconyx, Holmen, Wisconsin, USA) in locations that would maximize probability of detection based on local habitat conditions and known locations from collar data. We attempted to determine whether translocated individuals were travelling with other uncollared animals every 30 days by setting cameras for approximately 1 week, or until we were able to confirm target animals were travelling with other individuals or had left the area. All experimental methods were carried out with the approval of the University of Georgia’s Animal Care and Use Committee under protocol A2015 05004Y2A3 and were carried out in accordance with the ARRIVE guidelines for the immobilization of animals for studies conducted in the field. 

## Data analysis

### Space use metrics

Prior to analyses, we subsampled data from collars to 2-h intervals, the minimum common temporal resolution among animals used in this research. We also removed all locations with Position Dilution of Precision values > 9^42^. To evaluate effects of translocation, we calculated a suite of movement and space use statistics for resident and translocated pigs in both upland pine and bottomland hardwood habitats using a 7-day moving window analysis^24^. We first built the UD across the entire monitoring period for each animal, then used a moving window to incorporate each individual day as well as the 3 days prior and after. For example, day 4 would be included in calculations for days 1 through 7. Within the moving window framework, we calculated: (1) a 50% (hereafter, core area) and 95% (hereafter, range area) dynamic Brownian bridge movement model (dBBMM)^43^ utilization distribution, (2) Euclidian distance (m) to release site, and (3) daily distance (m) travelled for each pig. For all animals, we evaluated fidelity to the release site by calculating the daily mean distance from release site using the 12 GPS locations taken each day. For resident animals, we calculated distance to release site based on capture/release location. We calculated mean distance travelled for each individual by summing distance between each GPS location (i.e., step lengths) taken each day (hereafter, distance travelled) to evaluate daily movement rates^24^. For each metric, group (translocated or resident), and habitat type (bottomland hardwood or upland pine), we computed a mean and standard error across the 7-day periods for 90 days post release, or until the collar was retrieved, whichever was shorter. Three translocated wild pigs returned to their original home range within 10 days of translocation (Table S1). We considered animals returning to their original home range once they were within 1.6 km (radius of average resident home range size) of initial capture location. When this occurred, we removed data from the time of release until the animal had returned to their original home range and considered the individual as a resident at that time. We also censored three pigs after they each began travelling with another collared animal (Table S1). All analyses were conducted using program R (version 4.2.1) and dBBMM utilization distributions were calculated using the R package *move*^44^.

The dBBMM method requires a time-stamped series of locations and an estimate of telemetry error. We used an error estimate of 15 m for all locations based on vendor estimates. The dBBMM varies the Brownian motion variance (${\upsigma }_{m}^{2}$) for different subsections of the trajectory by moving a sliding window encompassing *n* number of locations along a path (see Kranstauber et al.^43^ for details). To fit the dBBMM, we specified a window size of 11 fix locations (equivalent to 22 h) and a margin of 5 fix locations based on the temporal resolution of each track and our a priori assumptions of the time-scale of behavioral breaks^43^.

### Resource selection

**Figure 1:**
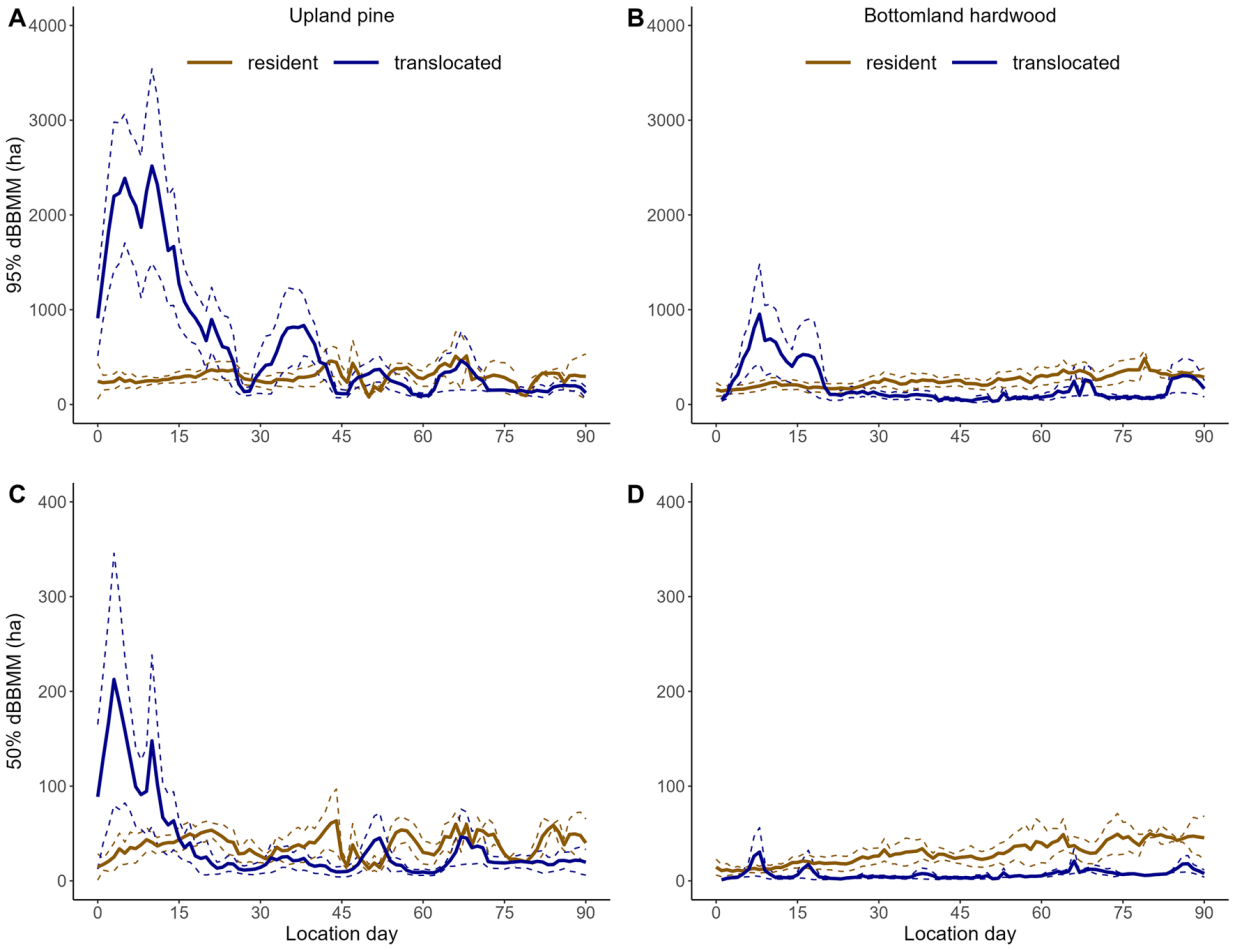
Dynamic Brownian bridge movement models at the 95% utilization distribution contour (**A** upland; **B** bottomland) and 50% contour (**C** upland; **D** bottomland) for resident and translocated wild pigs within a 7-day moving window on the Savannah River Site, SC, USA. Solid lines indicate means and dashed lines are ± 1 standard error. Note the Y axis not on same scale for both graphs.

To quantify resource selection, we used data from the National Land Cover Database (https://www.mrlc.gov/data) with a resolution of 30 × 30 m. We condensed the 15 habitat classifications to three coarse land cover types: bottomland (e.g., marsh, wetlands, bottomland hardwood, deciduous forest), upland pine (e.g. pine woodlands), and shrub/grassland (e.g. herbaceous and shrub/scrub) based on results of prior studies within our landscape^33,34^, which we assumed differed in provision of food and cover for wild pigs^31,33,45^. We excluded four other categories; developed (consisting of 4 levels), cultivated (consisting of 2 categories), open water, and barren land due to their limited distribution in and around the SRS site. To assess selection of these habitat types we used step selection functions (SSFs)^46^. Each step is compared to *n* number of random points drawn from a distribution of step lengths and turning angles. Thus, SSF’s constrain selected and available habitat types in both space and time. Animals introduced into novel locations are likely to exhibit different movement parameters when exploring versus exploiting new environments^24,47^, therefore, we used results from our moving window analysis to assign a distribution of step lengths and turning angles for generating random points based upon establishment phase (i.e., exploration vs. exploitation) and release habitat type (i.e., bottomland hardwood vs. upland pine). In addition, we created a single distribution of step lengths and turning angles for resident animals regardless of habitat type. To distinguish between exploration and exploitation for translocated individuals, we used results from our moving window analysis assessing 95% dBBMM use areas. We considered animals switching from exploration to exploitation once the 95% dBBMM for each translocated group reached the average for their respective resident group (see results; Fig. 1). On average, animals translocated to upland pine exhibited similar use areas (95% dBBMM; Fig. 1A) to residents on day 29, while those translocated to bottomland hardwood achieved similar patterns on day 22 (Fig. 1A). Consequently, we generated separate distributions for step length and turning angles for days 1–29 (exploratory) and days 30–90 (exploitation) for upland pine wild pigs, and for days 1–22 (exploration) and days 23–90 (exploitation) for bottomland hardwood wild pigs. As used area for resident wild pigs was similar between habitat types based on dBBMM (Fig. 1), we used one turning angle and step length distribution for both. We generated 20 random steps for each wild pig step using step lengths and turning angle distributions observed in this study while excluding the individual for which the distributions are being applied to^47^. Habitat type was recorded at the endpoint of each observed step. To reduce bias from animals being collared for long periods of time, we truncated all locations at 90 days or whenever the collar failed, whichever was shorter. GPS tracks were processed using the *AdehabitatLT* package in the R statistical Framework^48^.

To compare habitat types of used and available steps, we applied a conditional logistic regression with *amt*’s *fit_clogit* function^49^. Our first objective was to test whether resource selection differed between resident and translocated individuals, release habitat type, or the interaction of these factors. To examine this, we fit four models (Table 1A): (1) *habitat only* (3 habitat covariates and step length), (2) habitat*status (resident or translocated), (3) habitat*release habitat (dummy variable indicating whether the animal was released in primarily upland pine or bottomland hardwood habitat), and (4) habitat*status + *release* habitat. We also included a random effect term to account for individual variation and strata (strata term accounts for autocorrelation by grouping gps locations that are sequential and thus more likely to be correlated). We ranked all models using Akaike Information Criterion (AIC)^50^. Additionally, we performed a post-hoc test to assess whether resource selection differed between diel periods as previous research has indicated wild pigs are likely to exhibit different behaviors across the diel cycle^33^. We added an interaction term indicating whether the location was taken during diurnal or nocturnal time periods to our top-ranked model above. We considered models ≤ 2 AIC as equivalent^51^. Our second objective was to determine how selection for our hypothesized preferred habitat type (bottomland) differed through time based on release habitat type, and between resident and translocated individuals (Table 1B). For this analysis we estimated 7-day selection coefficients for bottomland habitat for each group based only on nocturnal locations as previous research has indicated female wild pigs tend to spend a higher proportion of time resting during diurnal hours^33^. We added a fixed effect for each 7-day period that an animal was on the air post release and calculated the average selection for bottomland hardwood for that group. We used a generalized linear mixed-effects model (GLMM) from the *lme4* package^52^ in R with a term to account for temporal autocorrelation.

**Table 1:** Candidate models and associated hypothesis tested to examine the influence of translocation status (resident or translocated) and habitat quality (good vs poor) on (A) overall resource selection patterns and (B) weekly selection for preferred (bottomland hardwood) habitat over 90 days post release.

(A) Models	Hypothesis
1. Habitat only	Resource selection (RS) only driven by habitat quality
2. Habitat * status	RS differs between resident and translocated animals regardless of habitat quality
3. Habitat * release habitat	RS differs by release habitat quality regardless of being translocated or residents of that area
4. Habitat * (status + release habitat)	RS differs based on habitat quality and whether the animal was translocated or resident

(B) Group	Hypothesis
5. Resident bottomland	Selection for preferred habitat (bottomland hardwood) will remain constant through time as animals have a ‘mental map’ of the environment
6. Resident upland	Selection for preferred habitat will remain constant
7. Translocated upland	Selection for preferred habitat will increase through time as wild pigs gain knowledge of the novel environment
8. Translocated bottomland	Selection for preferred habitat will increase at a slower rate than those translocated into lower quality (upland pine) habitats

## Results

We captured 31 female wild pigs and removed two animals from analysis due to capture-related mortality < 1 day post capture. We tracked the remaining 29 individuals from 14 to 209 days (mean = 103.6 day, SE = 10.7 day; Table S1), although as mentioned previously we truncated the data to 90 days post release for all analyses. Three females were initially released at the capture location then subsequently re-trapped and translocated, thus they were included in both resident and translocated datasets; the remaining individuals were included as either reference or translocated animals. On average, we released translocated animals 17.1 km (SD = 3.8 km) from their original capture location and monitored them for 113.6 days (range 14–209 days; Table S1). One translocated wild pig was harvested by a hunter outside of SRS 53 days after release and was censored at that time. In 2016, we obtained post-translocation sounder formation data from cameras for four females at 30 days and seven females at 60 days. Three of four (75%) translocated wild pigs were documented travelling with other conspecifics at 30 days, and six of seven (86%) at 60 days.

**Figure 2:**
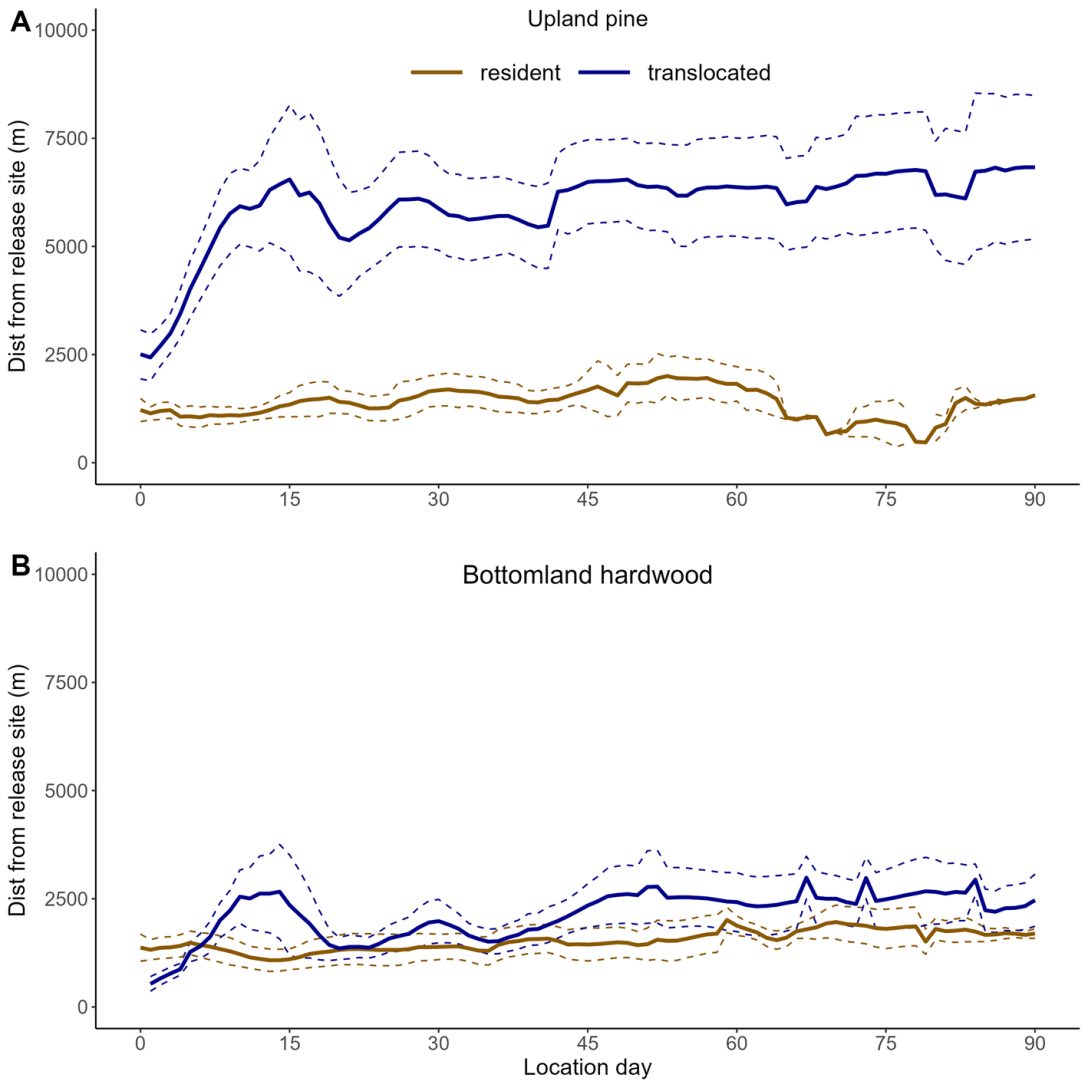
Distance from release site for resident and translocated wild pigs in (**A**) upland pine habitat and (**B**) bottomland hardwood habitat within a 7-day moving window on the Savannah River Site, SC, USA. Solid lines indicate means and dashed lines are ± 1 standard error.

Of the 26 radio-collared wild pigs that were included in our analyses, we classified 14 as residents (upland pine = 4; bottomland hardwood = 10) and 14 as translocated (upland pine = 8; bottomland hardwood = 6; Table S1); two individuals were captured initially as residents then subsequently recaptured and translocated. Overall, during the exploration phase, translocated individuals exhibited larger use areas, travelled further from their release site, and had larger daily movements than resident animals (Figs. 1, 2, Figure S1). Resident animals had relatively consistent range and core areas across the 90-day window (Fig. 1), and tended to remain within ~ 1 km of their release location (Fig. 2).

### Space use metrics

Wild pigs translocated from bottomland hardwood to upland pine habitat moved furthest from the release location (Figs. 1A, 2) and had the largest range and core areas, exhibiting two peaks in home range area on day five (2230.1 ha; SE = 604.5 ha) and day 10 (2,202.9 ha; SE = 934.8 ha; Fig. 1A). In contrast, ranges of individuals translocated from upland pine to bottomland hardwood habitat peaked on day seven (947 ha; SE = 524.02) at less than half the area of animals translocated from bottomland hardwood to upland pine habitat (Fig. 1B). Core areas followed a similar trajectory, although the magnitude was reduced for animals translocated to bottomland hardwood habitat (Fig. 1C,D). Range area for individuals translocated to upland pine was > 2 times that of those translocated to bottomland hardwood for both the exploration (upland pine = 1217.3 ha, SE = 121.7 ha; bottomland hardwood = 421.7 ha, SE = 50.6 ha) and exploitation phases (upland pine = 290.4 ha, SE = 20.3 ha; bottomland hardwood = 110.1 ha, SE = 8.0 ha); however, during the exploitation phase, both translocated groups had smaller use areas on average than resident animals from those habitat types (Fig. 1).

Distance to release site was consistent across resident animals. Among translocated wild pigs, individuals showed the greatest change in distance moved from the release site during the exploration phase before stabilizing during the exploitation phase (Fig. 2). Wild pigs translocated to upland pine dispersed furthest from release location, with the greatest rate of increase occurring from day one (2.4 km; SE = 0.5 km) to day 15 (6.5 km; SE = 1.7 km; Fig. 2A). Wild pigs translocated to bottomland hardwood followed a similar trajectory, although the magnitude was less; dispersal distance on day one was 0.7 km (SE = 0.2 km) and increased to 2.7 km (SE = 1.1 km) on day 13 (Fig. 2B). During the exploration phase (first 29 days for upland pine; 22 days for bottomland hardwood), range area for individuals translocated to upland pine (1,217.3 ha; SE = 121.7 ha) was > 4 times that of individuals translocated to bottomland hardwood habitat (290.4 ha; SE = 20.3 ha). All animals translocated to high quality bottomland hardwood habitat were residing in similar areas at the end of their respective monitoring periods. In contrast, 50% (n = 4) of animals translocated to upland pine dispersed to areas that were predominately bottomland habitat at the end of their monitoring periods.

### Resource selection

**Figure 3:**
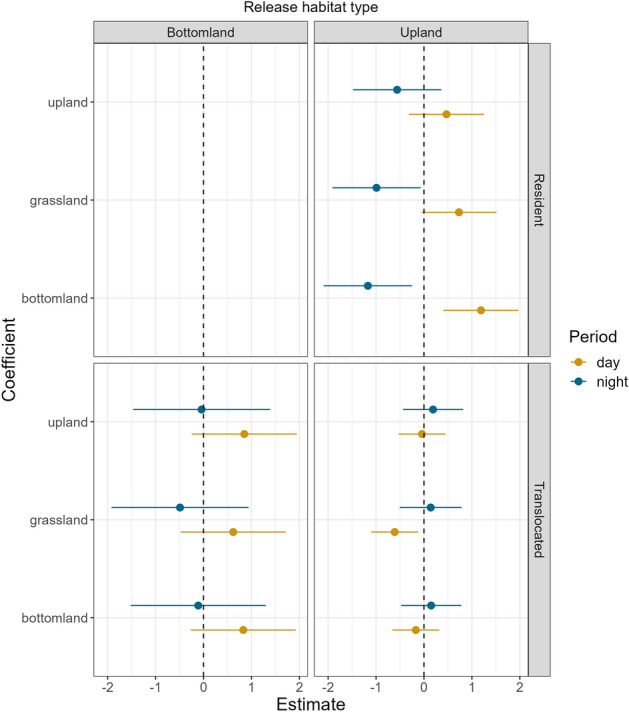
Standardized coefficients and 95% confidence interval from our top-ranked model for translocated and resident wild pigs on the Savannah River Site, SC, USA: model habitat × treatment × diel period. Intercept line (0) indicates neither selection nor avoidance. Estimates shown in relation to resident bottomland hardwood wild pigs.

Our analysis strongly supported the model incorporating both status (i.e., translocated or resident) and release habitat (bottomland hardwood or upland pine; Tables S2–S4). Despite the addition of 16 parameters, our post-hoc test accounting for diel period ranked higher than our previous top-ranked model, indicating wild pigs were exhibiting different nocturnal and diurnal selection patterns. The most significant changes in resource selection we observed were for wild pigs in low quality upland pine habitat. Residents of upland pine demonstrated avoidance of both grassland and bottomland habitat during nocturnal hours and selection for both habitat types during diurnal hours (Fig. 3). In contrast, animals translocated to upland pine showed significant avoidance of grassland habitat types during diurnal hours, and, though not significant, tended to exhibit greater selection for both grassland and bottomland habitat types during nocturnal hours (Fig. 3). Wild pigs translocated from upland pine to bottomland habitat exhibited no significant changes from their resident counterparts, although they did tend to select for all three habitat types more during diurnal hours than residents (Fig. 3).

### Nocturnal weekly resource selection

**Figure 4:**
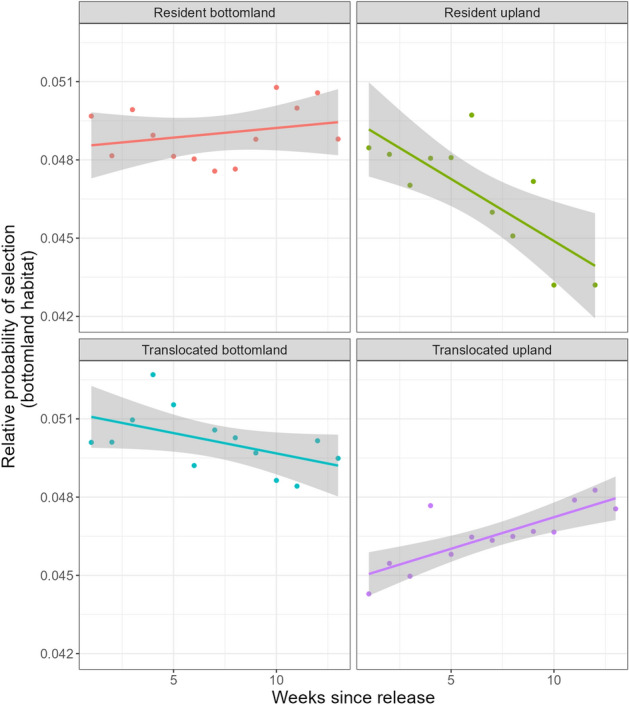
Nocturnal weekly selection coefficients for preferred habitat (bottomland hardwood) for translocated and resident wild pigs on the Savannah River Site, SC, USA.

Overall, each of our treatment groups exhibited varied selection for bottomland hardwood habitat over the 13-week period (Fig. 4). Both resident bottomland hardwood and resident upland pigs showed relatively little temporal change in selection, with resident upland animals exhibiting slightly lower selection overall. Wild pigs translocated to primarily bottomland habitat exhibited a decrease in selection of bottomland areas through time, while those translocated to upland habitat increased (Fig. 4).

## Discussion

Our results revealed differences in space use patterns of translocated wild pigs through time that were consistent with the exploration–exploitation hypothesis. Further, our data demonstrate habitat quality can mediate both the spatial and temporal scale at which animals respond behaviorally to novel environments as wild pigs introduced into high quality bottomland hardwood habitats displayed reduced exploratory periods and made less extensive movements than those introduced into lower quality upland pine habitats. Movement patterns of translocated animals, whether conservation-related, accidental, or intentional release of invasive or game species, may influence the success of population establishment as individuals that move greater distances or for longer periods of time may incur increased mortality risk and have a higher probability of emigrating from the release site^17,22^. Although, as resources become more abundant the need for an individual to explore their surrounding environment is reduced^9^.

Animals translocated to new landscapes will have incomplete knowledge of the environment; thus, developing a spatial representation of this new system through exploration requires a substantial expenditure of time and energy^53^. This increased energy output could result in reduced foraging time or have other fitness-related consequences. Indeed, longer acclimatization times have been associated with increased mortality. For instance, Frair et al*.*^17^ found site fidelity for translocated elk was directly related to forage resources encountered, and higher movement rates in low fidelity areas reduced elk survival. Similarly, Moehrenschlager and Macdonald^13^ found survival of swift foxes (*Vulpes velox*) was negatively correlated with distance translocated individuals moved from the release site. Longer exploratory bouts, both spatially and temporally, could also decrease reproductive success^13^, further reducing the founder population’s probability of establishment. For example, Poirier and Festa-Bianchet^54^ found translocated bighorn sheep required 1 year to acclimate and integrate into the local population. This delayed integration resulted in lower mass gain that ultimately led to a 1–2 year delay in reproduction, and those that did integrate more rapidly received more aggressive behavior (e.g. kicked, displaced).

In our study, we translocated only individual adult females that were not relocated with their social group, yet we suspect many illegal translocations could also involve groups of individuals to promote establishment of reproductive populations. Interestingly, we observed several instances of translocated wild pigs locating other translocated individuals or joining resident groups, reflecting the strong social dynamics of this species. Thus, it is possible group dynamics could affect the results we observed under scenarios involving group translocations. Group foragers such as wild pigs may be able to acquire social information from other conspecifics rather than solely relying on personal sampling^55–57^ to reduce some fitness-related costs associated with exploration of novel environments. For example, Clapp et al*.*^58^ documented a 19.5 day reduction in acclimation period from second and third releases of bighorn sheep compared with initial translocation efforts. However, although we did not test this directly, we observed three instances where two translocated wild pigs came into contact and began travelling together shortly after translocation and demonstrated similar movement patterns, both spatially and temporally, to those travelling alone. In fact, two of these individuals (135 and 136) were initially part of the same social group and were translocated separately, yet after rejoining had the largest movement rates of any translocated animals.

Results of SSF models using both resident and translocated animals indicate both extrinsic (e.g., habitat type) and intrinsic (e.g., translocated or resident) variables can influence wild pig resource selection, and highlights the adaptability of this invasive species. Distance to wetlands and bottomland habitat has been shown to be an important driver of wild pig movements^33,39^ and occurrence probability^59^, and was likely a key factor affecting resource selection and movement metrics in our study. Water sources are more abundant in bottomland hardwood habitats on our study site. Thus, the fact that wild pigs translocated to upland pine areas exhibited much lower selection for this feature, especially during the first few weeks post translocation, likely required these individuals to make larger movements through low-quality areas to meet basic physiological needs.

Selection for bottomland hardwood habitat generally supported our main hypotheses; (1) resident animals would exhibit relatively static selection through time, and (2) animals translocated to less suitable habitat (upland pine) would show a greater increase in selection of preferred habitat (bottomland hardwood) than those translocated directly into bottomland hardwoods as both groups developed a mental map of the area. Overall, wild pigs translocated to upland pine areas had the lowest selection for bottomland habitat during the first few weeks post translocation, suggesting initial movement behavior may have been focused attempting to relocate to their former range, but showed a positive trend over the 13 week monitoring period. Indeed, during the exploration phase, wild pigs tended to make relatively rapid straight-line movements followed by longer duration periods in which animals moved as if feeding or resting. Additionally, during the exploration phase wild pigs often made quick foray loops (e.g., < 1 day) after which the animal returned to a central location. These relatively quick-duration movements were possibly diluted in our analysis by the longer periods of time in these centralized locations. More fine-scale temporal resolution from GPS collars could elucidate whether animals are selecting for habitats differently during these quick duration exploratory movements, and warrants further investigation. It is also worth highlighting that at the end of the monitoring period, except for the resident upland group, the other treatment groups tended to converge near the same probability of selection for our hypothesized preferred habitat type. This might indicate some optimal amount of bottomland hardwood habitat is required to meet basic biological needs (e.g., as thermal refugia^31,33^).

Recent technological advancements in GPS telemetry equipment have facilitated a greater understanding of animal movement ecology, and provided the opportunity to understand how a variety of species respond to novel environments^13,22,44,58,60^. However, most studies have assessed movement dynamics from a conservation-oriented perspective and less attention has focused on how large invasive species respond to similar introductions into novel environments. Expansion of wild pig populations from illegal translocations by humans for hunting purposes has been well documented^27,28,61,62^, and our analysis indicates there is high dispersal potential from initial release sites prior to establishment within a relatively short time frame. These large-scale movements could hamper mitigation efforts aimed at removing newly established populations and increase risk of cross-species transmission of disease from wild pigs to wildlife, livestock, and humans^63^. Furthermore, more erratic movements during exploratory phases may increase risk to humans and property through vehicle collisions^38,64^. Based on our findings, these risks are likely to decrease temporally from initial release to acclimation and associated home range establishment and to be habitat dependent.

Regardless, predictions of an individual’s response to translocation can provide important insight into risks associated with these activities and facilitate more effective translocation strategies for species of conservation concern, e.g.,^10^. Indeed, nearly 30% of conservation translocation efforts are unsuccessful^4^ and a primary reason translocation efforts fail is long-distance or frequent movements exhibited by released animals^65,66^, which may increase the animal’s probability of mortality or emigration from the site^20^. Similarly, invasive species can greatly affect ecosystems and cause extirpation or extinction of native species^67,68^, and knowledge of the movement ecology of animals in novel environments may allow improved mitigation of risks posed by invasive animals following their introduction. However, there is a need for additional information regarding how group dynamics may affect the process of exploratory behavior and acclimation to novel environments, as well as the long-term consequences of translocation on overall fitness in both occupied and unoccupied habitats^60,69^. Thus, we recommend future research address these knowledge gaps to facilitate more effective strategies for the application of species introductions and translocations as potential management and conservation tools, as well as to better mitigate threats from introductions of invasive species.

Currently our understanding of how individuals integrate multiple sources of information to make decisions regarding where to establish is underdeveloped. While there is a substantial body of literature characterizing existing home ranges, using translocations we can observe the process of home range development. This can contribute to improved understanding of higher order processes governing not only home ranges but of species and individuals assorting across the landscape. Detailed tracking of translocated individuals also can shed light on the processes of exploration and settlement, and ultimately help link population-level fitness with space use. Thus, continued expansion of our understanding of underlying processes contributing to the successful establishment of species within diverse landscapes remains critical to the refinement of policies and strategies governing the conservation and management of species.

## Supplementary Information

Supplementary Information.

## Data Availability

The datasets used during the current study are available from the corresponding author on reasonable request.
