# Supplementary material for: Habitat quality influences trade-offs in animal movement along the exploration–exploitation continuum
Source: Sci Rep. 2023 Mar 24;13:4814. doi: 10.1038/s41598-023-31457-3 (PMC10039022; doi:10.1038/s41598-023-31457-3)

Supporting Information. Joshua B. Smith, David A. Keiter, Steven J. Sweeney, Ryan S. Miller, Peter E. Schlichting, James C. Beasley. Habitat quality influences trade-offs in animal movement along the exploration-exploitation continuum. *Scientific Reports*.

Table S1. Status and duration of monitoring for 32 sows captured on the Savannah River Site, SC, USA.

| Status | ID | Release date | End date | n (days) | Translocation distance (km) | Release habitat |
| --- | --- | --- | --- | --- | --- | --- |
| resident | 127 | 6/12/2014 | 7/28/2014 | 46 | na | UP |
| resident | 132 | 1/22/2015 | 7/30/2015 | 189 | na | BH |
| resident | 135^1.2^ | 2/9/2015 | 4/1/2015 | 51 | na | BH |
| resident | 136^2^ | 2/9/2015 | 4/1/2015 | 51 | na | BH |
| resident | 137 | 4/15/2015 | 6/12/2015 | 58 | na | BH |
| resident | 140 | 3/4/2015 | 4/8/2015 | 35 | na | BH |
| resident | 142 | 4/23/2015 | 6/12/2015 | 50 | na | BH |
| resident | 143^2^ | 4/20/2015 | 7/7/2015 | 78 | na | BH |
| resident | 144 | 4/21/2015 | 11/13/2015 | 206 | na | BH |
| resident | 652 | 6/17/2014 | 7/28/2014 | 41 | na | UP |
| resident | 167 | 2/16/2016 | 8/14/2016 | 180 | na | UP |
| resident | 169 | 2/19/2016 | 8/19/2016 | 182 | na | UP |
| resident | 146^3^ | 7/9/2015 | 9/25/2015 | 78 | 15.8 | BH |
| resident | 211^3^ | 4/29/2016 | 7/21/2016 | 83 | 19.9 | BH |
| resident | 220^3^ | 7/1/2016 | 9/2/2016 | 63 | 12.9 | BH |
| translocated | 129 | 2/5/2016 | 7/18/2016 | 164 | 19.8 | BH |
| translocated | 135^2^ | 4/1/2015 | 6/7/2015 | 67 | 16.0 | UP |
| translocated | 136 | 4/1/2015 | 6/11/2015 | 71 | 18.9 | UP |
| translocated | 143 | 7/7/2015 | 8/29/2015 | 53 | 19.8 | UP |
| translocated | 149 | 6/12/2015 | 1/7/2016 | 209 | 8.2 | UP |
| translocated | 150 | 6/19/2015 | 1/7/2016 | 201 | 19.0 | UP |
| translocated | 153 | 7/7/2015 | 9/25/2015 | 81 | 19.7 | UP |
| translocated | 157 | 8/17/2015 | 1/7/2016 | 143 | 19.3 | UP |
| translocated | 158^1^ | 2/29/2016 | 3/24/2016 | 24 | 19.3 | BH |
| translocated | 159 | 1/30/2016 | 7/19/2016 | 171 | 20.3 | BH |
| translocated | 160 | 1/30/2016 | 7/21/2016 | 173 | 19.0 | BH |
| translocated | 201 | 3/5/2016 | 7/21/2016 | 138 | 18.4 | UP |
| translocated | 207 | 3/21/2016 | 7/6/2016 | 107 | 8.5 | BH |
| translocated | 209 | 3/28/2016 | 6/8/2016 | 72 | 12.7 | BH |
| translocated | 210^1^ | 3/28/2016 | 7/19/2016 | 113 | 14.3 | BH |
| translocated | 213 | 5/18/2016 | 9/17/2016 | 122 | 19.5 | UP |
| translocated | 216 | 6/14/2016 | 6/28/2016 | 14 | 19.8 | BH |

^1^Was not included in analysis as sample was not independent – travelled with another translocated sow.

^2^Sows were monitored in situ and subsequently re-trapped and translocated to elucidate pre- and post-translocation movements.

^3^Sows were translocated but made their way back to original capture location. Sows were excluded from translocated animals, and we considered them residents after returning to initial capture area.

Table S2. Step selection models for resident and translocated wild pigs (*Sus scrofa*) on the Savannah River Site, SC, USA ranked using Akaike Information Criterion (AIC). K = number of parameters; w = model weight; ΔAIC is the difference between the AIC of the current model to that with the lowest AIC value.

| Model | K | AIC | ∆AIC | *w_i_* |
| --- | --- | --- | --- | --- |
| ^1^treatment * period | 31 | 84065.5 | 0 | 0.93 |
| ^2^treatment | 15 | 84061.7 | 5.2 | 0.07 |
| ^3^status | 7 | 84096.0 | 39.5 | 0 |
| ^4^rel hab | 7 | 84163.2 | 106.7 | 0 |
| habitat only | 3 | 84184.0 | 127.5 | 0 |

^1^ post-hoc model added to assess whether wild pigs exhibited temporal differences in selection between diurnal and nocturnal locations.

^2^ treatment = term specifying whether wild pigs were released as residents or translocated, and specifying release habitat type (i.e., bottomland hardwood or upland pine).

^3^ Status = resident or translocated.

^4^ Release habitat = upland pine or bottomland hardwood.

Table S3. Model summary statistics from top-ranked model (treatment * period).

| Test | Value | *P* |
| --- | --- | --- |
| Likelihood ratio test | 398.4 | <0.005 |
| Wald test | 378.2 | <0.005 |
| Log rank test | 384.8 | <0.005 |
| Concordance | 0.542 (±0.003) |  |

Table S4. Model outputs from top model (treatment * period).

| Covariate | Estimate | Exponent | SE | Z | P |
| --- | --- | --- | --- | --- | --- |
| Bottomland | 0.34 | 1.41 | 0.23 | 1.49 | 0.14 |
| Upland | 0.00 | 1.00 | 0.23 | 0.00 | 1.00 |
| Grassland | 0.29 | 1.34 | 0.23 | 1.25 | 0.21 |
| Night | 0.16 | 1.18 | 0.29 | 0.55 | 0.58 |
| Resident upland | -0.71 | 0.49 | 0.39 | -1.83 | 0.07 |
| Translocated bottomland | -0.78 | 0.46 | 0.56 | -1.41 | 0.16 |
| Translocated upland | 0.27 | 1.31 | 0.24 | 1.15 | 0.25 |
| Bottomland * Night | -0.19 | 0.83 | 0.30 | -0.63 | 0.53 |
| Upland * Night | -0.26 | 0.77 | 0.30 | -0.85 | 0.40 |
| Grassland * Night | -0.05 | 0.95 | 0.30 | -0.17 | 0.87 |
| Resident upland * Night | 0.89 | 2.43 | 0.45 | 1.98 | 0.05* |
| Translocated bottomland * Night | 0.16 | 1.17 | 0.72 | 0.22 | 0.83 |
| Translocated upland * Night | -0.13 | 0.87 | 0.31 | -0.44 | 0.66 |
| Bottomland * Resident upland | 1.19 | 3.30 | 0.40 | 3.01 | 0.00* |
| Bottomland * Translocated bottomland | 0.83 | 2.30 | 0.56 | 1.49 | 0.14 |
| Bottomland * Translocated upland | -0.17 | 0.84 | 0.25 | -0.71 | 0.48 |
| Upland * Resident upland | 0.47 | 1.60 | 0.40 | 1.18 | 0.24 |
| Upland * Translocated bottomland | 0.85 | 2.34 | 0.56 | 1.51 | 0.13 |
| Upland * Translocated upland | -0.04 | 0.96 | 0.25 | -0.18 | 0.85 |
| Grassland * Resident upland | 0.73 | 2.08 | 0.40 | 1.85 | 0.06 |
| Grassland * Translocated bottomland | 0.62 | 1.85 | 0.56 | 1.10 | 0.27 |
| Grassland * Translocated upland | -0.61 | 0.54 | 0.25 | -2.44 | 0.01* |
| Bottomland * Resident upland * Night | -1.17 | 0.31 | 0.47 | -2.49 | 0.01* |
| Bottomland * Translocated bottomland * Night | -0.11 | 0.90 | 0.72 | -0.15 | 0.88 |
| Bottomland * Translocated upland * Night | 0.15 | 1.16 | 0.32 | 0.46 | 0.65 |
| Upland * Resident upland * Night | -0.56 | 0.57 | 0.47 | -1.20 | 0.23 |
| Upland * Translocated bottomland * Night | -0.04 | 0.96 | 0.73 | -0.05 | 0.96 |
| Upland * Translocated upland * Night | 0.19 | 1.21 | 0.32 | 0.60 | 0.55 |
| Grassland * Resident upland * Night | -0.99 | 0.37 | 0.47 | -2.13 | 0.03* |
| Grassland * Translocated bottomland | -0.49 | 0.61 | 0.73 | -0.67 | 0.50 |
| Grassland * Translocated upland * Night | 0.14 | 1.15 | 0.33 | 0.42 | 0.68 |

* Denotes significant value.

Figure S1. Daily distance traveled for resident and translocated wild pigs in (A) upland pine habitat and (B) bottomland hardwood habitat within a 7-day moving window on the Savannah River Site, SC, USA. Solid lines indicate means and dashed lines are ±1 standard error.


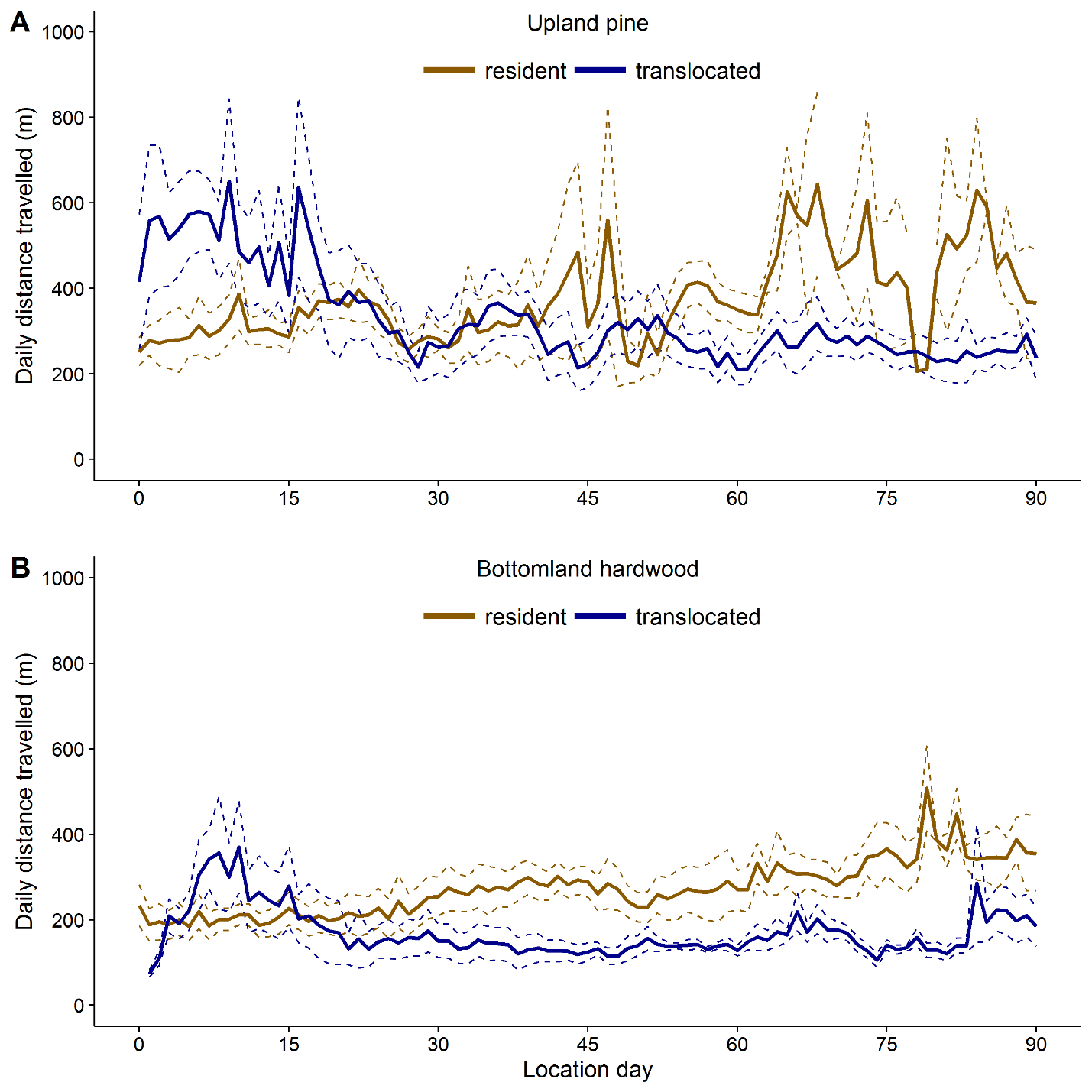

Supplement: Supplementary file 1 — Supplementary Information. [file 41598_2023_31457_MOESM1_ESM.docx]
